# RPGR protein complex regulates proteasome activity and mediates store-operated calcium entry

**DOI:** 10.18632/oncotarget.25259

**Published:** 2018-05-01

**Authors:** Sarita Rani Patnaik, Xun Zhang, Lincoln Biswas, Saeed Akhtar, Xinzhi Zhou, Deva Krupakar Kusuluri, James Reilly, Helen May-Simera, Susan Chalmers, John G. McCarron, Xinhua Shu

**Affiliations:** ^1^ Department of Life Sciences, Glasgow Caledonian University, Glasgow G4 0BA, Scotland; ^2^ Institute of Molecular Physiology, Johannes Gutenberg-Universität Mainz, D-55128 Mainz, Germany; ^3^ Cornea Research Chair, Department of Optometry, King Saud University, Riyadh 11433, Kingdom of Saudi Arabia; ^4^ Strathclyde Institute of Pharmacy and Biomedical Sciences, University of Strathclyde, Glasgow G4 0RE, Scotland

**Keywords:** ciliopathy, RPGR complex, actin cytoskeleton, endoplasmic reticulum, store-operated Ca^2+^ entry

## Abstract

Ciliopathies are a group of genetically heterogeneous disorders, characterized by defects in cilia genesis or maintenance. Mutations in the *RPGR* gene and its interacting partners, *RPGRIP1* and *RPGRIP1L*, cause ciliopathies, but the function of their proteins remains unclear. Here we show that knockdown (KD) of *RPGR, RPGRIP1* or *RPGRIP1L* in hTERT-RPE1 cells results in abnormal actin cytoskeleton organization. The actin cytoskeleton rearrangement is regulated by the small GTPase RhoA via the planar cell polarity (PCP) pathway. RhoA activity was upregulated in the absence of RPGR, RPGRIP1 or RPGRIP1L proteins. In *RPGR, RPGRIP1* or *RPGRIP1L* KD cells, we observed increased levels of DVl2 and DVl3 proteins, the core components of the PCP pathway, due to impaired proteasomal activity. *RPGR, RPGRIP1* or *RPGRIP1L* KD cells treated with thapsigargin (TG), an inhibitor of sarcoendoplasmic reticulum Ca^2+^- ATPases, showed impaired store-operated Ca^2+^ entry (SOCE), which is mediated by STIM1 and Orai1 proteins. STIM1 was not localized to the ER-PM junction upon ER store depletion in *RPGR, RPGRIP1* or *RPGRIP1L* KD cells. Our results demonstrate that the RPGR protein complex is required for regulating proteasomal activity and for modulating SOCE, which may contribute to the ciliopathy phenotype.

## INTRODUCTION

Primary cilia are microtubule-based, non-motile, solitary organelles emerging from the surface of many vertebrate cells. During early stages of ciliogenesis the centriole docks at the apical membrane and becomes the basal body; the subsequent axoneme is generated by microtubule extension and is surrounded by a specialized ciliary membrane forming primary cilia [1]. Genetic defects result in abnormal cilia formation or function leading to a variety of disorders collectively called ciliopathies [1], which include Bardet-Biedl syndrome (BBS), Joubert syndrome (JS) and Meckel syndrome (MKS). Defects in cilia function can also result in non-syndromic diseases such as retinitis pigmentosa (RP). RP is a group of heterogeneous neurodegenerative diseases characterized by the death of photoreceptors. X-linked RP (XLRP) is the most severe form of RP, accounting for 10-20% of all RP cases [2]. Mutations in the retinitis pigmentosa GTPase regulator (*RPGR*) gene are the major cause of XLRP [3–5]. Previous work has shown that RPGR interacts with RPGR-interacting protein-1 (RPGRIP1) [6–8] and RPGRIP1-like protein (RPGRIP1L) [9, 10]. Defects in RPGRIP1 cause a form of severe congenital retinal dystrophy (Leber congenital amaurosis, LCA), juvenile RP and cone-rod dystrophy [11–13]. Loss-of-function mutations in the *RGRIP1L* gene led to either JS or the lethal MKS [14, 15]. In mouse, deletion of RPGR results in a slower retinal degeneration [16, 17], while loss of RPGRIP1 leads to an early onset retinal degeneration with abnormal development of outer segments [18, 19]. Retinal degeneration has also been reported in dogs carrying naturally occurring mutations in the *RPGR* or *RPGRIP1* gene [20, 21]. Morpholino-induced knockdown of *RPGR* in zebrafish results in ciliary defects and abnormal retinal development [22]. Global deletion of RPGRIP1L in mouse causes mid-gestation lethality with cilia defects in multiple organs, corresponding closely to the clinical phenotype observed in MKS [14]. Together these data suggest that RPGR, RPGRIP1 and RPGRIP1L are critical in ciliary homeostasis.

Indeed, RPGR and RPGRIP1 have been reported to co-localize in the connecting cilia of photoreceptors and centrosomes/basal bodies of differentiating cells [8, 18, 23, 24]. RPGRIP1L is also localized to the basal bodies of ciliated cells and of cilia in renal tubules, retina and brain [14, 15, 25]. RPGR forms a protein complex with RPGRIP1, RPGRIP1L and other ciliary proteins including NPHP1, NPHP4, CEP290, SPATA7 and NEK4 [26]. Our previous work has demonstrated that knockdown of RPGR in hTERT-RPE1 cells resulted in impaired ciliogenesis and cell attachment, stronger actin filaments and abnormal focal adhesion, suggesting RPGR functions in cilia formation and regulation of actin dynamics [27]. To gain further insight into the function of RPGR and its interactors (RPGRIP1 and RPGRIP1L) and to understand the underlying mechanisms of action, we used RNA-interference-mediated translational suppression (knockdown, KD) strategy in the hTERT-RPE1 cell model and studied the signal transduction pathways involved. We found that loss of RPGR, RPGRIP1, or RPGRIP1L caused remodeling of the actin cytoskeleton. We also observed upregulation of RhoA- GTPase activity, increased levels of DVL2/3 and impaired store-operated Ca^2+^ entry (SOCE) in RPGR, RPGRIP1 or RPGRIP1L KD cells. We provide compelling evidence that RPGR, RPGRIP1 and RPGRIP1L may function in ciliopathy by regulating the activity of proteasome and mediating SOCE.

## RESULTS

### Loss of RPGRIP1 or RPGRIP1L causes RhoA-mediated actin cytoskeleton defect

It has been reported that RPGR KD resulted in stronger actin filaments in hTERT-RPE1 cells [27].

**Figure 1:**
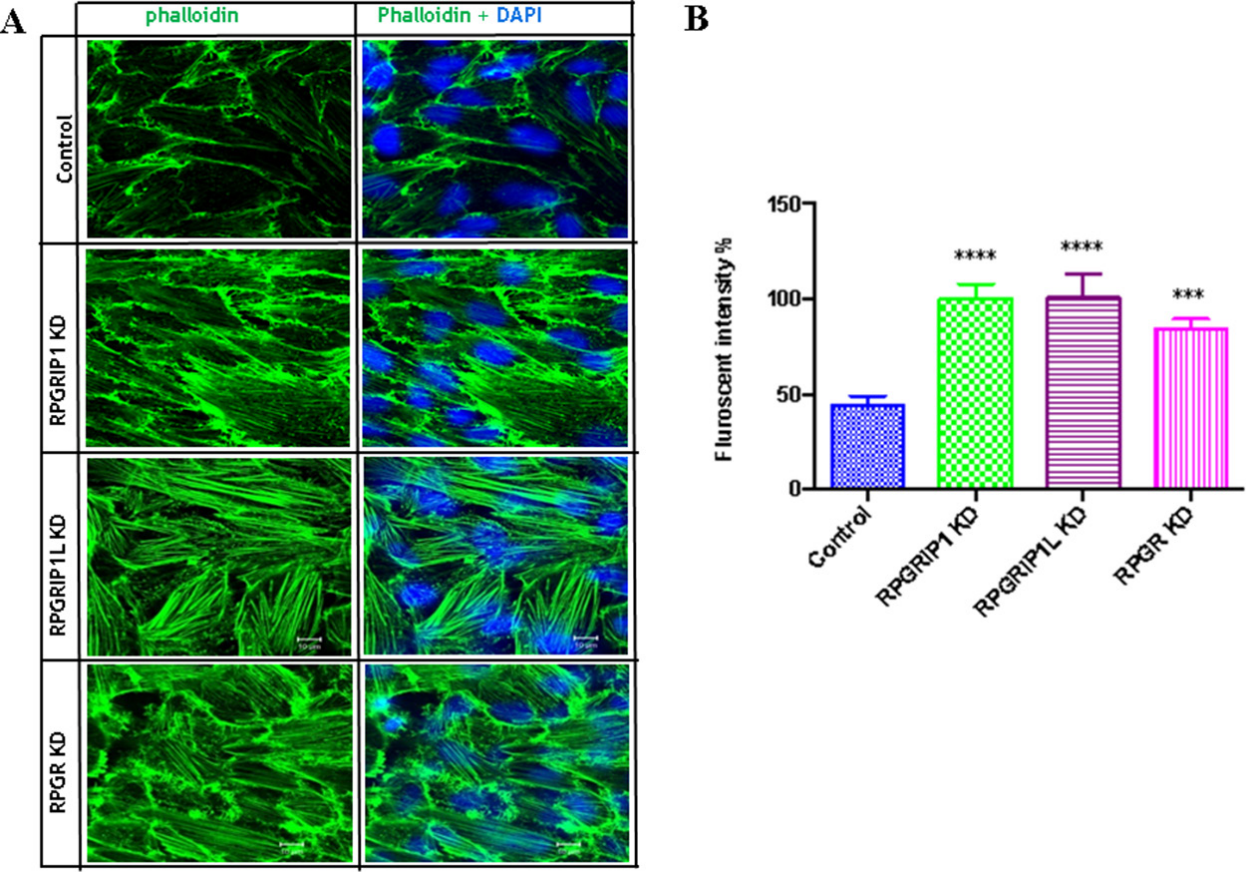
RPGRIP1, RPGRIP1L or RPGR knockdown (KD) cells showed stronger actin filaments **(A)** hTERT-RPE1 cells were transfected with control or RPGR, RPGRIP1 or RPGRIP1L specific siRNAs. Forty eight hours after transfection cells were stained with FITC-conjugated phalloidin (green). DAPI was used to visualize nuclei. **(B)** Actin filament fluorescence intensity in control and KD cells as measured by image J software. Florescence signals were from three individual slides for each KD cell. When compared to the scrambled control cells, RPGR, RPGRIP1 or RPGRIP1L KD cells had significantly higher florescence signals. Data were presented as means±SEM. Statistical significance was analyzed using one-way ANOVA test followed by Dunnett’s test. ^***^p<0.001; ^****^p<0.0001.

**Figure 2:**
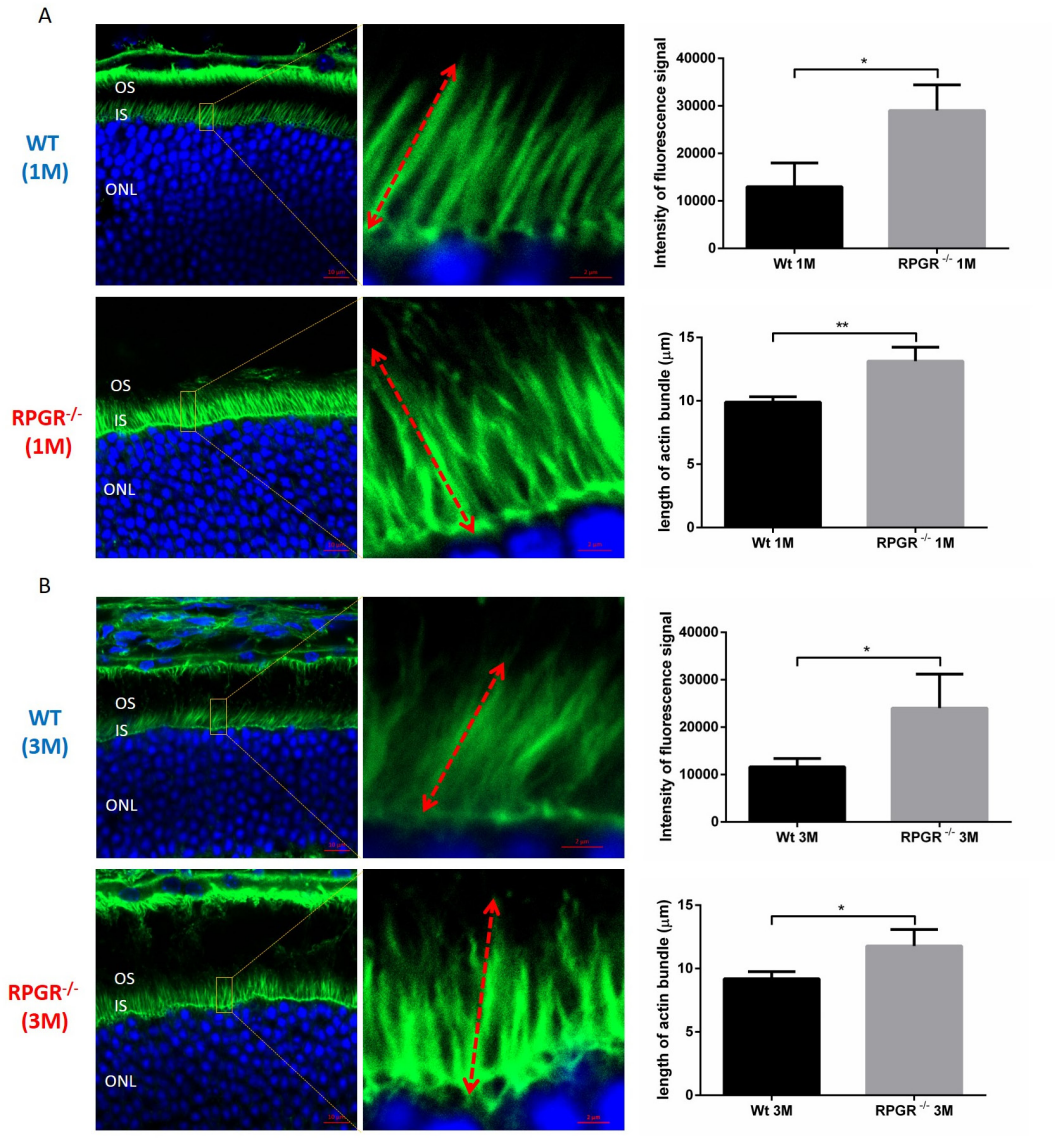
Cryosections of 1 month **(A)** and 3 months **(B)** old wildtype (WT) and RPGR knockout (KO) mouse retinal sections were stained with phalloidin-FITC to show the actin bundles in photoreceptors. DAPI was used for the visualization of photoreceptor nuclei. In each retinal section, the areas for the measurement of actin bundle length were made at 300μm to the optic nerve head (superior side). The intensity of fluorescence signal of FITC and the length of actin bundle were measured by ZEN software. Actin filaments were significantly stronger and the actin bundles were significantly longer in the photoreceptors of RPGR KO mice when compared to that of wildtype mice. Graphs represent intensity of average fluorescence signal per 10×10μm^2^ area (three areas from three individual mouse eye sections) stained with phalloidin and average length of actin bundles, respectively. Measurements of 5 actin bundles were made within each of three randomly selected areas; in total, three sections, each from a different mouse eye, were examined, providing measurements from 45 bundles. Double-head arrow represents the full-length of actin bundle. IS, inner segment; ONL, outer nuclear layer; OS, outer segment. Data were presented as means±SEM. Statistical significance was analysed using one-way ANOVA followed by by Dunnett’s test. ^*^P<0.05; ^*^ P<0.01.

To examine the role of RPGR, RPGRIP1 or RPGRIP1L in regulation of the actin cytoskeleton, we used small interfering RNAs (siRNAs) to deplete RPGR, RPGRIP1 and RPGRIP1L in hTERT-RPE1 cells. Quantitative real-time PCR (qRT-PCR) and Western blotting were performed at 48 h post transfection to verify the efficiency of RPGR, RPGRIP1 or RPGRIP1L depletion and confirmed that the three genes were effectively knocked down (Supplementary Figure 1). We examined the cytoskeleton in RPGRIP1 or RPGRIP1L depleted cells. We used FITC-phalloidin to label F-actin and found that denser actin stress fibers were observed in *RPGRIP1* or *RPGRIP1L* KD cells 48 h after transfection (Figure 1A, 1B). Similarly, we also depleted RPGR in hTERT-RPE1 cells and checked for the expression of actin stress fibers. As reported, actin filaments were increased in *RPGR* KD cells (Figure 1A, 1B) [27]. Although the precise morphology of the stress fibers varied somewhat between the different conditions, there was a noticeable increase in actin density compared to scrambled control. We also used a biochemical approach (described in Materials and Methods) to fractionate F-actin and G-actin in control and KD cell lysates and found a significant increase in F-actin to G-actin ratio in *RPGR, RPGRIP1* and *RPGRIP1L* KD cells when compared to that of control cells (Supplementary Figure 2). Next we examined actin in the photoreceptors of *RPGR* knockout mice at one and three months old by phalloidin-FITC staining; we found that the actin signal in *RPGR* knockout photoreceptors was significantly stronger than that of wildtype mice at both ages (Figure 2). We also measured the length of the actin bundles in the photoreceptors in these mice and found that they were longer in *RPGR* knockout mice than in wildtype control mice (Figure 2A, 2B).

Having previously reported a cilia defect in RPGR-knockdown hTERT-RPE1 cells [27], we now examined whether RPGRIP1- or RPGRIP1L-depleted cells also displayed a cilia defect. Immunofluorescence microscopy revealed that RPGRIP1 or RPGRIP1L siRNA-mediated KD cells had a significantly lower number of ciliated cells compared with the non-targeting siRNA transfected control cells, as determined by staining with anti-ARL13B (axoneme marker) and anti-GT335 (basal body/transition zone marker) antibodies (Supplementary Figure 3). We also found significantly fewer ciliated cells in RPGR KD cells, consistent with the findings of Gakovic *et al* [27]. These results showed that RPGR, RPGRIP1 or RPGRIP1L is required for cilia formation. Since the ciliogenesis assay was performed after serum starvation, we further analysed the formation of actin upon serum starvation. The KD cells (RPGRIP1, RPGRIP1L or RPGR) were serum starved and stained with FITC-phalloidin. We visualized Z-stack images using confocal microscopy. In the control cells the actin was reorganized cortically while in KD cells actin stress fibers were distributed throughout the cells (Supplementary Figure 4). Particularly in RPGRIP1 or RPGRIP1L KD cells there is an increase in ventral stress fibers, which were severely distorted and disorganized (Supplementary Figure 4).

**Figure 3:**
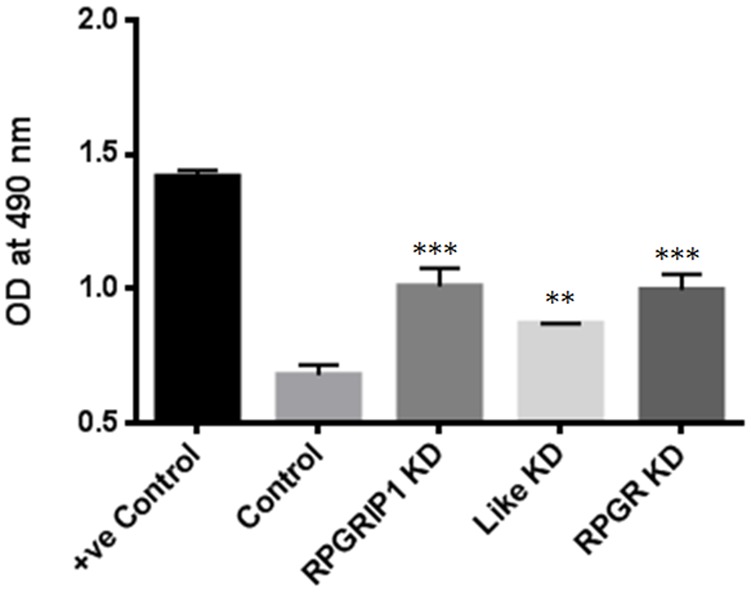
RhoA activities in RPGRIP1, RPGRIP1L or RPGR deficient cells Significantly increased level of active RhoA-GTPases was observed in RPGR, RPGRIP1, or RPGRIP1L knockdown (KD) cells. The first column (+ve control) is a positive control for the experiment, provided in the kit. When compared to nonspecific RNAi transfected (scrambled control) cells, RPGR, RPGRIP1, or RPGRIP1L KD cells displayed significant increase in RhoA-GTP activities. This experiment was repeated three times. Data were presented as means±SEM. Statistical significance was analyzed using one-way ANOVA test followed by Dunnett’s test. ^**^p<0.01; ^***^p<0.001.

Planar cell polarity (PCP) signaling is implicated in governing actin architecture [28]. RhoA family members of small GTPases are key mediators of the PCP pathway [29]. Increased RhoA has also been shown to increase F-actin polymerization and to regulate actin dynamics [29–31], and has also been shown to be up-regulated in other cilia mutant cell lines [32]. We therefore used an enzyme-linked immunosorbent assay to determine the levels of RhoA-GTP in these KD cells. We observed that RhoA is hyper-activated in the KD cells compared to the non-targeting siRNA transfected control cells (Figure 3) These results clearly demonstrated that hyperactivation of RhoA was the underlying cause of increased actin stress fiber formation.

### DVL proteins are increased in KD cells due to impaired proteasome

**Figure 4:**
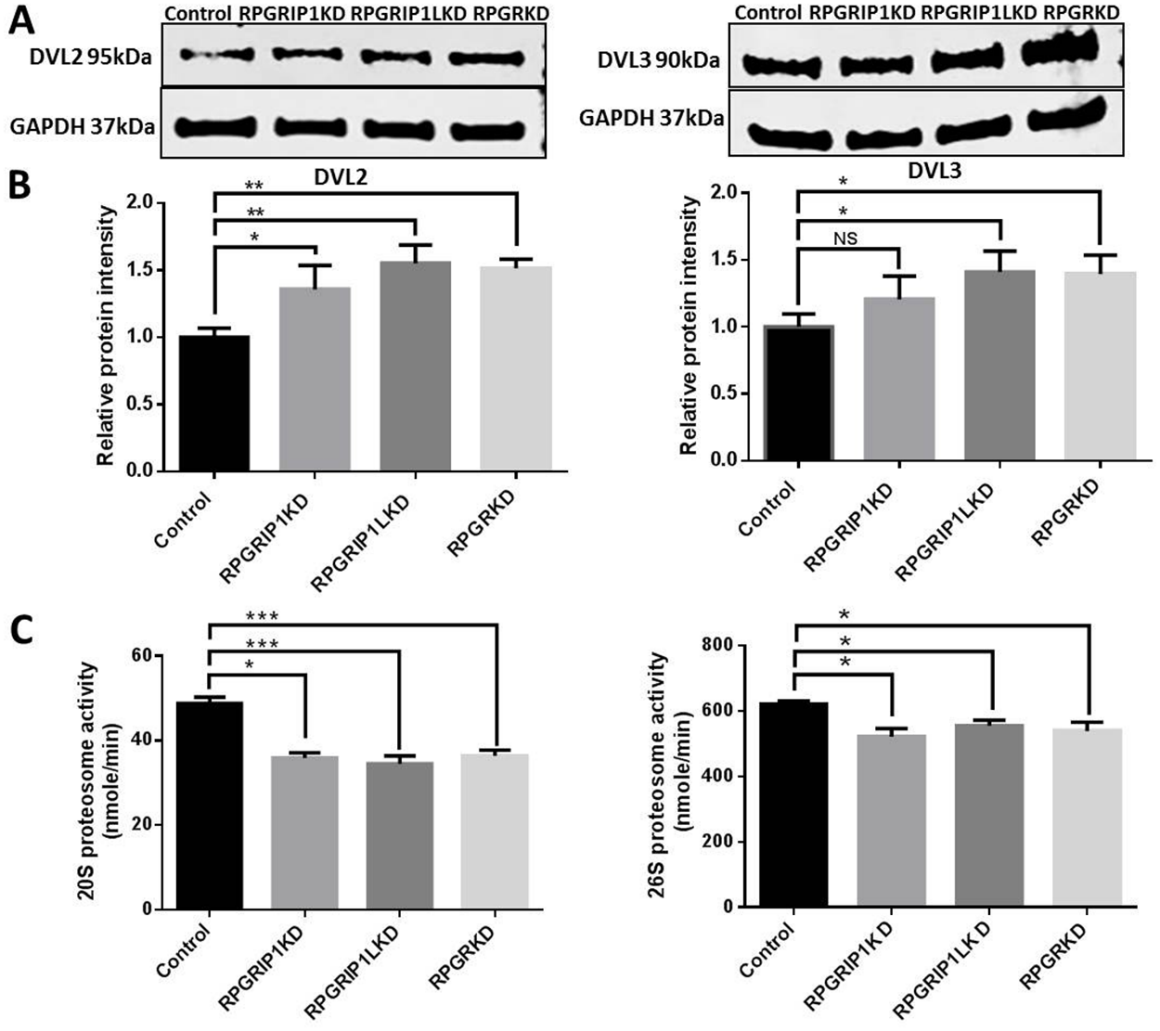
Significantly increased levels of DVL2 and DVL3 in RPGRIP1, RPGRIP1L or RPGR Knockdown (KD) cells due to proteasomal defect **(A)** DVL2 and DVL3 in control and KD cell lysates were detected by Western blot using anti-DVL2 and anti-DVL3 antibodies. The blots were reprobed with GAPDH as loading control. **(B)** Graph represents the relative band intensity of DVL2 and DVL3 in scrambled control and KD cells. The band intensities of DVL2 and DVL3 were measured and normalized with the band intensity of GAPDH. The ratio of DVL2 or DVL3 to GAPDH in scrambled control cells was regarded as 1.0. **(C)** Proteasomal activity was measured in total cell lysates of control hTERT-RPE1 and KD cells. There was a significant reduction of proteasome activity in both ATP independent (20S) and dependent (26S) proteasome compared to that of scrambled control cells. The experiment was repeated three times. Data were presented as means±SEM. Statistical significance was analyzed using one-way ANOVA test followed by Dunnett’s test. NS, no significance; ^*^p<0.05; ^**^p<0.01; ^***^p<0.001.

Wnt activation of RhoA requires the cytoplasmic protein Dishevelled (DVL) [33]. DVL-associated activator of morphogenesis 1 (*Daam1*) binds to both RhoA and DVL thereby forming the complex that activates the cell polarity signal transduction and mediates cytoskeletal reorganization [33]. To examine whether DVL protein was increased in *RPGR, RPGRIP1* or *RPGRIP1L* KD hTERT-RPE1 cells, we performed western blot to analyze the levels of DVL2 and DVL3. We observed a significant increase in DVL2 and DVL3 protein in *RPGR, RPGRIP1* or *RPGRIP1L* KD hTERT-RPE1 cells (Figure 4A, 4B). A previous report demonstrated that knockdown of NPHP4, an interactor of RPGRIP1 and RPGRIP1L, resulted in decreased levels of DVL2 and DVL3 in MDCK cells [34]. The authors suggested that NPHP4 targeted DVL2 and DVL3 for proteasomal degradation. Accordingly, we investigated the proteasomal function in control and RPGR, RPGRIP1 or RPGRIP1L KD cells by measuring 20S and 26S proteasomal activities. We found both 20S and 26S proteasomal activities were significantly decreased in KD cells when compared to control cells (Figure 4C). We also treated hTERT-RPE1 cells with MG132, a proteasome inhibitor, which effectively blocks the proteolytic activity of the 26S proteasome complex, and found that the levels of DVL2 and DVL3 were markedly increased in these MG132-treated cells (Supplementary Figure 5), suggesting that DVL2 and DVL3 were degraded by the proteasome.

### RPGRIP1, RPGRIP1L or RPGR KD cells show decreased store-operated Ca^2+^ entry

**Figure 5:**
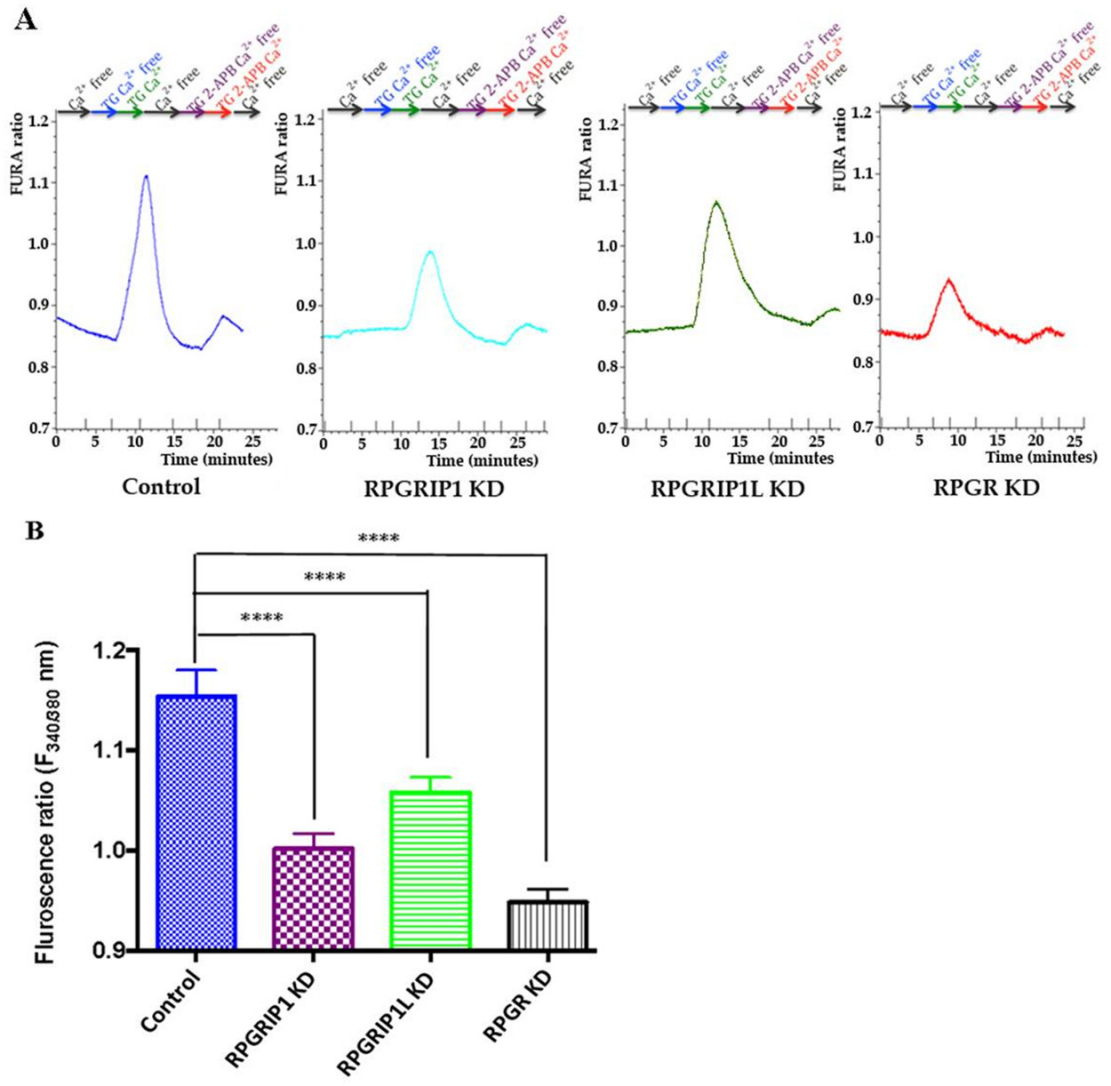
SOCE in control or RPGR, RPGRIP1, or RPGRIP1L knockdown (KD) hTERT-RPE1 cells **(A)** Cells in ringer solution were first washed with Ca^2+^ free solution until the resting Ca^2+^ stabilized. Following this, cells were treated with thapsigargin (TG, 1μM) in Ca^2+^ free solution for 5 minutes, then Ca^2+^ was reintroduced along with TG for 5 min followed by a Ca^2+^ free wash. Next the cells were treated with 2-APB and TG in Ca^2+^ free solution followed by application of 2-APB (75 μM, inhibitor of SOCE) and TG in presence of Ca^2+^ and finally washed with Ca^2+^ free solution. The SOCE is decreased in RPGRIP1 KD, RPGRIP1L KD and RPGR KD cells compared to the scrambled control cells on application of TG. **(B)** Comparison of SOCE between control and KD cells using one-way ANOVA test followed by Dunnett’s test. 14 cells were analyzed in each experiment. Three independent experiments were performed per group. ^****^p<0.0001

Store-operated Ca^2+^ entry (SOCE) is a ubiquitous Ca^2+^ signaling mechanism that plays an important role in many cellular processes [35]. The actin cytoskeleton plays a modulatory role in SOCE in various cell types [36]. Since depletion of RPGR, RPGRIP1, or RPGRIP1L affects actin remodeling, we investigated whether the three proteins regulate cellular Ca^2+^ signaling by modulating SOCE. Live cell intracellular Ca^2+^ measurement was done using Fura2/AM, an intracellular Ca^2+^ indicator. Cells were first bathed with Ca^2+^-free extracellular solution and then exposed to 3μM thapsigargin (TG, a sarco/endoplasmic reticulum Ca^2+^-ATPase (SERCA) inhibitor) in Ca^2+^-free solution, which ensures depletion of the endoplasmic reticulum (ER) Ca^2+^ stores. Thapsigargin alone did not increase Ca^2+^, presumably since the ER had been depleted rapidly in the Ca^2+^-free bathing solution. Subsequently, Ca^2+^ was re-added, to enable the SOCE response following Ca^2+^ store depletion, which leads to a transient increase in intracellular Ca^2+^ influx. We observed that KD of RPGRIP1 attenuated the TG-induced Ca^2+^ entry. Similarly, KD of RPGRIP1L or RPGR also resulted in decreased SOCE (Figure 5). We used 75μM 2-Amino-ethoxydiphenylborate (2-APB), a SOCE inhibitor [37], to suppress Ca^2+^ entry and found that there was no significant difference in the basal level of Ca^2+^ between the control and KD cells. Activation of cell surface receptors leads to the generation of the signalling molecule inositol 1,4,5-trisphosphate (IP_3_), which in turn results in a rapid release of Ca^2+^ from ER via the IP_3_ receptor (IP_3_R) [35]. To investigate the role of these proteins in IP3-mediated (inositol trisphosphate) Ca^2+^ release from the ER Ca^2+^ store, further experiments were performed using muscarinic receptor agonist Carbachol (CCh; 10 μM), an IP3-generating agonist that is a nonhydrolyzable analogue of acetylcholine. IP3-induced Ca^2+^ release from the store was not altered in KD hTERT-RPE1 cells compared to control, as the peak cytosolic Ca^2+^ evoked by CCh in all four cell types was similar (Supplementary Figure 6). Together these results suggest that neither RPGRIP1 nor RPGRIP1L or RPGR is involved in directly regulating ER Ca^2+^ release, although they participated in SOCE in hTERT-RPE1 cells. The ER content is similar presumably because of the time provided for ER refilling to occur (Supplementary Figure 6).

### RPGR, RPGRIP1 or RPGRIP1L KD causes loss of STIM1 localization to ER-PM junction

**Figure 6:**
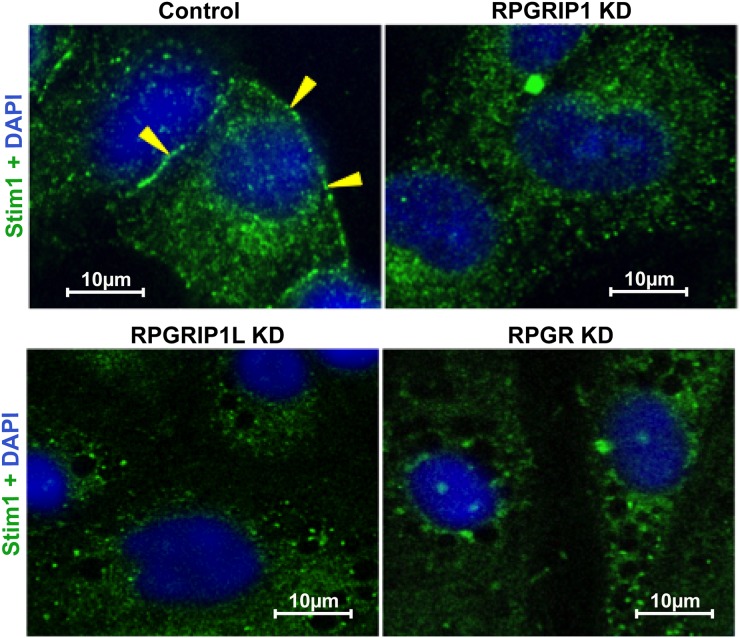
Defect in STIM1 localization to the ER-PM junction in knockdown (KD) cells RPGR, RPGRIP1 or RPGRIP1L KD hTERT-RPE1 cells were treated with thapsigargin (1μM) and immediately fixed and stained with STIM1 antibody. Prominent localization of STIM1 is observed in ER-PM (arrow-head) scrambled control cells but not in that of the KD cells.

Stromal interaction molecule 1 (STIM1), an ER Ca^2+^ sensor, is a single-pass transmembrane protein and localized throughout the ER. ORAI calcium release-activated calcium modulator 1 (Orai1) is a structural component of the Ca^2+^ release-activated Ca^2+^ (CRAC) channel. In response to ER Ca^2+^ store depletion, STIM1 and Orai1 migrate and co-localize to the ER-plasma membrane (ER-PM) junction, where STIM1 binds to Orai1 to open the CRAC channel [35]. We investigated whether ER Ca^2+^ depletion in KD cells involved any defect in the localization of STIM1 to ER-PM junction. As above, thapsagargin treatment was used to cause depletion in *RPGR, RPGRIP1* or *RPGRIP1L* KD and control cells; localization of STIM1 was then examined by immunostaining using an anti-STIM1 antibody. In control cells, a clear increase in STIM1 staining was observed at the ER-PM as expected. However we observed a defect in STIM1 localization to the ER-PM junction in KD cells which may explain the reduced SOCE (Figure 6).

## DISCUSSION

In spite of numerous studies, the pathological mechanisms underlying ciliopathies are still not clear. Ciliopathies show a broad range of genetic heterogeneity and overlapping phenotypes involving cross talk between various signal transduction pathways [38]. Different signaling pathways such as canonical and non-canonical Wnt [28, 39–40], Notch [41], Hedgehog [42], mTOR [43], and Hippo [44] have been implicated in ciliogenesis. The non-canonical Wnt signaling pathway (PCP) controls various biological processes such as cell migration, differentiation, polarity establishment, actin cytoskeletal rearrangement and cell survival [45-47]. DVL proteins are major actors in the PCP pathway [33]. DVL signals are mediated through a Daam1-RhoA axis, thus RhoA is the central factor of the PCP pathway and essential for the regulation of the actin cytoskeleton [33, 48]. Our current study demonstrated that RPGR, RPGRIP1 and RPGRIP1L regulate the DVL family of proteins and influenced the PCP pathway. Knock-down of RPGR, RPGRIP1 or RPGRIP1L caused an increase in DVL2 and DVL3 proteins (Figure 4A, 4B); it is possible that proteasome degradation was impaired in knock-down cells which led to more DVL2 and DVL3 protein being retained, since both 20S and 26S proteasomal activities were markedly decreased (Figure 4C). When hTERT-RPE1 cells were treated with MG132, the proteasome inhibitor that blocks 26S proteasomal function, the amount of DVL2 and DVL3 was significantly increased (Supplementary Figure 5). In support of this, Burcklé *et al* (2011) reported that treatment of MDCK cells with clasto-lactacystin, a selective inhibitor of the 20S proteasome, caused significantly increased levels of DVL2 and DVL3 [34]. Combined, these data suggest that endogenous DVL2 and DVL3 are degraded through the 20S and 26S proteasome pathways. Interestingly, Rao *et al* (2015) reported that Psmd3 (a proteasomal component of 19s subunit) was significantly reduced in photoreceptor outer segments of RPGR knockout mice when compared to the wildtype mice [49]. Recently Gerhardt *et al* showed that RPGRIP1L interacted with Psmd2 protein (a proteasomal component of 19s subunit) and that deletion of RPGRIP1L caused impaired proteasomal activity and decreased protein degradation [50]. However, Mahuzier *et al* found that knock-down of RPGRIP1L in MDCK cells resulted in a reduction of DVL2 and DVL3 proteins and suggested RPGRIP1L stabilized DVL proteins by preventing their proteasomal degradation [51]. The difference between our current data and the work presented by Mahuzier *et al* may reflect different underlying molecular mechanisms between hTERT-RPE1 and MDCK cells. Other ciliopathy proteins including BBS1,2,4,6,7,8, NPHP5 and OFD1 also interact with proteasomal components, while loss of BBS4, BBS7 and OFD1 also resulted in a decrease of proteasomal activity [52]. Knock-down of BBS proteins exhibited abnormal actin cytoskeleton organization and increased RhoA activity, suggesting similar underlying mechanisms [32]. Actin filaments have been observed along the photoreceptor axoneme, at the base of outer segments (OS), associated with the OS plasma membrane [53], and are involved in photoreceptor protein trafficking and in coordinating OS morphogenesis [54, 55]. RPGR knockout mice exhibited noticeably disorganized disc membrane at the base of photoreceptor OSs [16, 56]. Via Western blotting, Rao *et al* recently showed a 2-fold increase in the ratio of F-actin to G-actin and a significant increase in RhoA-GTP in *RPGR* KO retinas [56]. Together with our data showing abnormal actin cytoskeleton in hTERT-RPE1 cells and in the photoreceptors of RPGR KO mice (Figure 1 and 2), this suggests a role of the RPGR protein complex in regulating the actin cytoskeleton, which may underlie the pathogenesis involved in retinal ciliopathies [57].

The actin cytoskeleton plays an important role in SOCE. It has been shown that disruption of the actin cytoskeleton using Cytochalsin D or latrunculin A significantly decreased SOCE in platelets, endothelial cells, type I astrocytes, glioma C6 cells and pancreatic acinar cells [36, 58, 59]. By contrast, inhibition of actin polymerization in NIH 3T3, DDT1MF-2 or A7r5 cells did not modify SOCE [36]. The ER is the main store for Ca^2+^ in nearly all metazoan cells; the Wnt/Ca^2+^ signaling pathway leads to ER Ca^2+^ release. Wnt5a (or other Wnt) binding to cognate Frizzled receptors trigger the activation of phospholipase C (PLC) and produces inositol 1,4,5-trisphophate (IP3) through phosphatidyl inositol 4,5-bisphophate hydrolysis by PLC. IP3 interacts with and opens a calcium channel, the IP3 receptor, in the ER membrane, resulting in the release of Ca^2+^ from ER store into the cytosol [35, 60]. The Ca^2+^ content in the ER is maintained via SOCE and the activity of STIM1. STIM1 continuously moves through the ER and is associated with the end binding protein 1 (EB1). Upon ER store depletion STIM1 dissociates from EB1 and translocates to the ER-PM junction [61], where it oligomerizes, forms puncta and associates with Orai1 thereby enabling Ca^2+^ influx into the cell and result in SOCE [35]. Septins regulate the recruitment of Orai1 at the PM where it interacts with STIM1 at the ER-PM junction [62, 63]. Septins are GTP-binding proteins that are recognized as cytoskeletal components and regulate actin dynamics [64]. Septin 2 is required for the ciliary membrane protein distribution and depletion of Septin 2 inhibits ciliogenesis [65]. Further investigation into Orai1, Septins and their relation with ciliopathy proteins, e.g. RPGR and its interacting proteins, will shed light on the underlying mechanisms of ciliopathies. Our data showed here that knockdown of RPGR, RPGRIP1, or RPGRIP1L in hTERT-RPE1 cells leaded to abnormal actin cytoskeleton through the PCP pathway and resulted in decreased SOCE (Figure 5). However knockdown of RPGR, RPGRIP1, or RPGRIP1L did not affect ER Ca^2+^ release (Supplementary Figure 5), suggesting that the RPGR protein complex is possibly involved in the PCP pathway but not the Wnt/Ca^2+^ pathway.

Ciliary proteins have been predicted to contain several distinct types of C2 domain, which interact with other functional domains and mediate important functions like cytoskeletal interactions, vesicular trafficking and organelle movement [66]. The C2C-domain of RPGRIP1 and RPGRIP1L is closely homologous to the C2 domain of protein kinase Cε (PKCε) [66]. The PKC-C2 participates in Ca^2+^ dependent membrane localization during various signaling processes and vesicle trafficking. PKC is also involved in the regulation of store operated Ca^2+^ influx to sustain long lasting Ca^2+^ oscillations in fertilized eggs [67]. Most of the C2 domains act as Ca^2+^ sensors by binding to Ca^2+^ or phospholipids [68]. However, it has been reported recently that Ca^2+^ doesn’t bind to the C2 of RPGRIP1 [10]. Therefore, the decrease in SOCE may not be due to an effect at the Ca^2+^ binding site but due to a defect in the actin cytoskeleton causing defective trafficking of STIM1. The ER is a very dynamic organelle that requires a cytoskeleton for its continuity, integrity and sliding dynamics. Since a cytoskeleton defect is the common denominator in RP (ref 56 and our current study), MKS [69] and BBS [32], it would be interesting to study ER dynamics in depth in additional ciliopathies.

**Figure 7:**
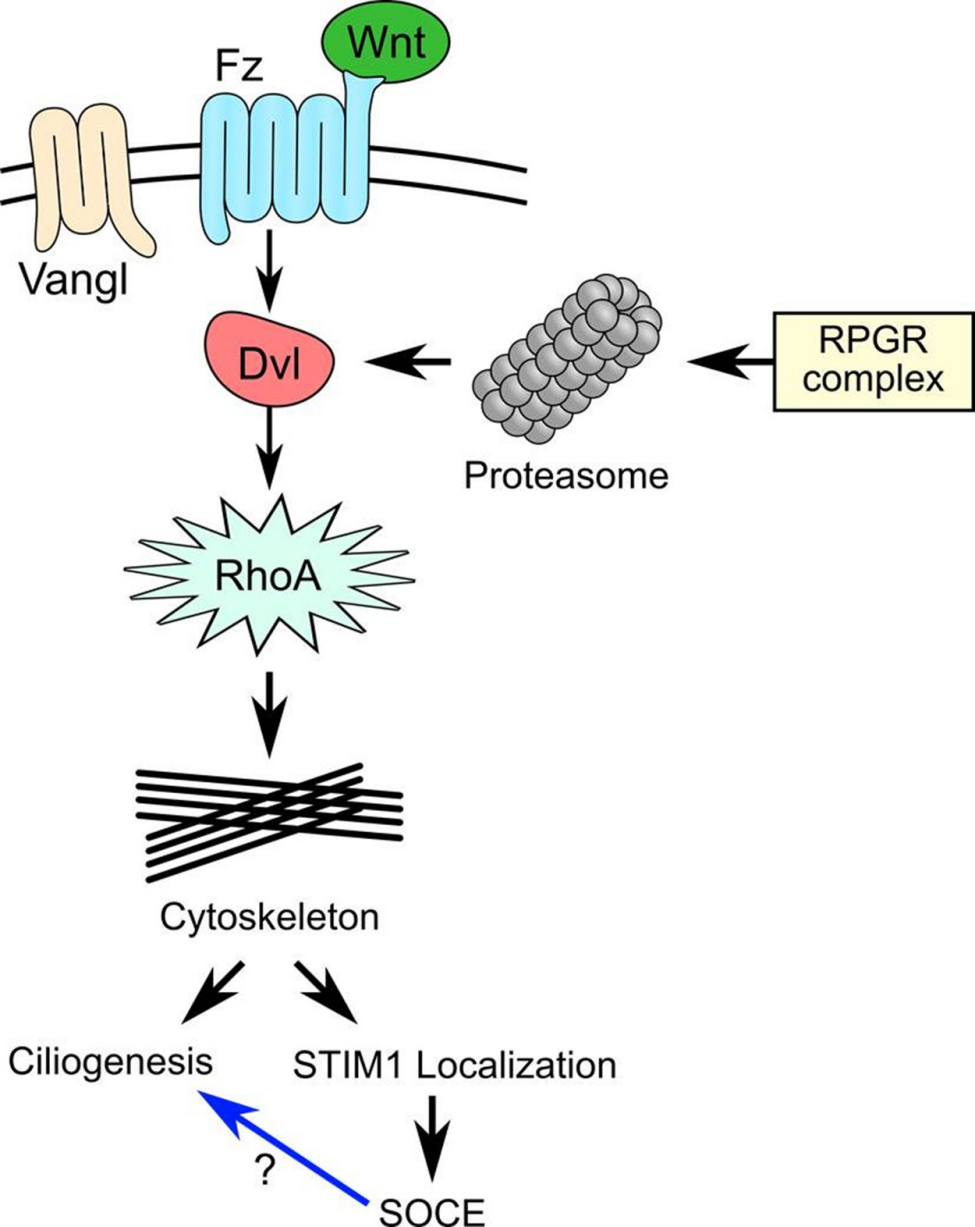
The RPGR protein complex is associated with the planar cell polarity (PCP) pathway Wnt ligands bind to Frizzled and its co-receptor (Vangl) and activates the PCP pathway, leading to the recruitment of Dishevelled (Dvl) and formation of a protein complex with Dishevelled-associated activator of morphogenesis 1 (DAAM1). DAAM1 then activates the small G-Protein, RhoA and regulate the cytoskeleton. The RPGR protein complex controls DVL degradation by regulating proteasomal activity. Loss of RPGR protein complex function resulted in decreased proteasomal activity and increased DVL protein, which upregulated RhoA and caused abnormal actin remodeling. Abnormal actin remodeling could directly lead to cilia defects or decreased store-operated Ca^2+^ entry (SOCE) due to loss of STIM1 ER-PM localization. It is not clear whether decreased SOCE can directly lead to cilia defect in RPE1 cells.

Our results demonstrate a fundamental role of the RPGR protein complex in regulation of actin dynamics through the involvement of the PCP pathway by regulating the activity of proteasome and mediating SOCE (Figure 7). Further studies will be required to elucidate the role of these proteins in the signaling pathways that mediate ciliopathies.

## MATERIALS AND METHODS

### Cell culture and transfection

hTERT-RPE1 cells were maintained in DMEM-F12 (LONZA), supplemented with 10% foetal bovine serum (FBS), sodium bicarbonate and penicillin/streptomycin. Cells were transfected with Lipofectamine 2000 (Life technologies) according to the manufacturer’s instructions. Briefly, cells were plated at a density of 1 X 10^5^ cells per well on a 12-well plate (Greiner Bio-One) the day prior to transfections. For immunostaining experiments cells were seeded on coverslips. One hour before transfection, growth medium was replaced by medium without antibiotics. Scrambled control and RPGR/RPGRIP1/RPGRIP1L specific siRNAs were diluted in OptiMEM (Gibco). 1.5μl of Lipofectamine-2000 was diluted in OptiMEM and incubated at room temperature (RT) for 5min. Both the diluents were mixed, incubated for 20 min, added drop wise to the cells and then incubated.

### Antibodies and siRNAs

The following antibodies were used for immunostaining: Phalloidin-FITC labelled: 50 μg/ml (P5282, Sigma), anti-STIM1 antibody (1:200, cell signalling technology), anti-ARL13B (1:200, Abcam), anti-GT335 antibody (1:200, Sigma), anti-DVL2 (1: 2000) (cell signalling technology, 3224P), anti-DVL3 antibodies (1: 2000) (cell signalling technology, 3218P), anti-RPGR antibody (1:500, Sigma), anti-RPGRIP1 antibody (1:500, Abcam), and GAPDH (1: 2000) (Fitzgerald Industries), were used for western blotting. All siRNAs were ordered from IDT, including scrambled control, RPGRIP1 (HSC.RNAI.N020366.12), RPGRIP1L (HSC.RNAI.N015272.12), RPGR (HSC.RNAI.N001034853.12).

### Immunofluorescence microscopy

For immunofluorescence analyses, cells were cultured on glass coverslips. Cells were fixed with ice-cold methanol at -20°C for 3 min, washed with PBS, then blocked with 2% BSA/PBS for 30 min at RT. Cells were then incubated with primary antibodies for 1-2h at room temperature, washed with PBS and blocked with 2% sheep serum in 2% BSA/PBS for 30 min at RT. Cells were washed with PBS followed by incubation with secondary antibodies for 45 min at RT in dark, then washed several times with PBS and mounted using Vectashield mounting media with DAPI (4,6-diamidino-2-phenylindole; Vector laboratories) to stain nuclei. Fluorescence signals were detected using Zeiss LSM510 confocal microscopes. For staining F-actin, cells were fixed with 4% PFA for 10 minutes at RT before blocking. All other steps were performed as mentioned above.

The *RPGR* knockout mouse model was gifted from Professor Wright’s group at MRC Human Genetics Unit, Edinburgh, UK. The *RPGR* knockout mouse model contains a deletion of the proximal promoter and first exon of the *RPGR* gene, resulting in loss of RPGR protein. The RPGR knockout mice demonstrated progressive photoreceptor degeneration and loss of visual function [17]. All experiments using mice were carried out in accordance with the UK home office animal care guidelines and approved by Glasgow Caledonian University Animal Ethics and Welfare Committee (Project licence number P8C815DC9); a minimal number of animals were used for this study. The enucleated eyes from both wildtype and RPGR knock-out mice were fixed in 2% PFA/PBS at 4°C for 18-24 hours. Then the eyes were dehydrated through the 5%, 15% and 20% sucrose (4 hours at each concentration) and embedded in Optimal Cutting Temperature compound (OCT). The 10 μm sections were air dried at room temperature for 10 min and rehydrated by wash buffer (1×TBS/0.025% Triton X-100) twice for 5min. Then the sections were incubated with blocking buffer (1×TBS/0.3% Triton X-100)/5% sheep serum) for 1 hour at room temperature and incubated with diluted Phalloidin-FITC (50 μg/ml, Sigma, UK) in blocking buffer overnight at 4°C. Sections were washed by wash buffer 5 times for 5min and counterstained with DAPI. The fluorescence of sections was photographed and analyzed by Zeiss LSM 800. The intensity of fluorescence signal of the actin branch was measured by ZEN software (Zeiss, Germany) within 10×10μm^2^ of the actin branch area from 3 sections of each mouse eye sample (four mouse eye samples were used). The length of the actin branch was similarly measured by ZEN software.

### Quantitative real-time PCR (qRT-PCR)

48 h after transfection, total RNA was extracted from cells using TRIzol reagent (Invitrogen) and approximately 1μg of RNA was reverse transcribed to cDNA using Transcriptor High Fidelity cDNA synthesis kit (Roche) according to manufacturer’s instructions. The resulting cDNA was used to perform qRT-PCR using SYBR Green PCR master mix (Life technologies) with an iCycler Real- Time PCR Detection System (BioRad). Transcript levels of target genes were normalized to transcript levels of GAPDH, which was used as a reference gene. Primer sequences for qRT-PCR will be provided when requested.

### RhoA assay

The levels of active GTP-bound RhoA protein in lysates prepared from RPGRIP1, RPGRIP1L or RPGR KD cells was determined by enzyme-linked immunosorbent assay (ELISA) using RhoA G-LISA^™^ activation assay kit (BK124, Cytoskeleton), which can detect the level of GTP-loaded RhoA only. Cell lysates were equalized for total protein concentrations and the measurement was performed according to manufacturer’s instruction.

### Measurement of proteasome activity

Control and RPGR, RPGRIP1 or RPGRIP1L KD cells were collected and washed twice with PBS. Cells were lysed by adding the lysis buffer (250 mM sucrose, 25 mM Hepes, 10 mM magnesium chloride, 1 mM EDTA, and 1.7 mM DTT) and going through repeated freeze–thaw cycles. The 20S proteasome activity (ATP independent) and 26S proteasome activity (ATP dependent) were measured with an assay in the absence of ATP (depleted by incubating with 0.1 mg/ml hexokinase and 15 mM 2-deoxyglucose (Sigma-Aldrich)) and one in the presence of ATP (in which 2mM ATP was added to the cells). Thereafter, cell lysates were incubated with 225 mM Tris buffer, pH 7.8, containing 45 mM potassium chloride, 7.5 mM magnesium acetate, 7.5 mM magnesium chloride, 1 mM DTT and the fluorogenic peptide suc-LLVY-AMC (7-amino-4-methylcoumarin; Sigma-Aldrich) for 30 minutes at 37°C. The fluorogenic peptide is degraded by the chymotrypsin-like protease of the proteasome into the degradation product, 7-Amino-4-methylcoumarin (AMC) (Sigma-Aldrich). The AMC was quantified by FluroStar OPTIMA with 380 nm excitation and 460 nm emission. For standard quantification, free AMC was used in different concentrations.

### Measurement of F-actin and G-actin

The control and RPGR, RPGRIP1 and RPGRIP1L KD cells were washed with PBS twice and then homogenized in F-actin stabilization buffer (50 mm piperazine-N, N′-bis(2-ethanesulfonic acid) at pH 6.9, 50 mM NaCl, 5 mM MgCl2, 5 mM ethylene glycol tetra-acetic acid, 5% glycerol, 0.1% NP40, 0.1% Triton X-100, 0.1% Tween 20, 0.1% β-mercaptoethanol, 1 mm adenosine triphosphate, and protease inhibitor cocktail) and incubated at 37°C for 10 minutes. After that, the cells were centrifuged at 100 000 × g for 1 hour. The supernatant was separated immediately into another tube and labelled as the G-actin fraction. The pellet was resuspended in ice cold water containing 10 μM cytochalsin D and incubated on ice for 60 min with brief mixing every 10 min. The samples were then sonicated and centrifuged at 13000 rpm for 10 minutes and the supernatant was collected for F-actin fraction. The G- and F-actin fractions were analyzed by immunoblotting. Fold change was calculated considering relative band intensity of the control sample.

### Western blotting

Cells were lysed using M-PER mammalian protein extraction reagent (Thermo Scientific) containing a protease inhibitors cocktail (Roche). The lysates were centrifuged at 4°C for 20 min at 16000g and supernatants were collected. Total protein concentration was determined using Precision Red advanced protein assay reagent (ADV02, Cytoskeleton). The supernatants were incubated for 5 min at 95°C with NuPAGE^®^ LDS sample buffer (NP007, Life Technologies). Equal amounts of protein were loaded and resolved using precast gels from Bio-rad (Mini PROTEAN TGX Gel, Cat number 456-9034) and electro-transferred to nitrocellulose membranes (Hybond, GE Healthcare). The blots were blocked with 5% milk powder and incubated with primary antibody according to the manufacturer’s protocol. Secondary antibody conjugated with horseradish peroxidase (HRP) was used for detection using ECL plus western blotting detection reagent (GE Healthcare). To confirm equal protein loading, all the immunoblots were also probed with anti GAPDH antibody. The immunoblots were scanned on the LI-COR Odyssey FC Imaging System and the signal intensity was analyzed by Image Studio™ Lite analysis software (LI-COR).

### Ca^2+^ imaging

Control or knockdown cells were loaded with 3 μM Fura-2 AM in Ringer solution for 45 min at 37°C. Cells were washed with Ringer solution and coverslips mounted onto bath chambers. They were superfused with Ringer solution and mounted onto the stage of the microscope. Radiometric measurements with Fura-2 AM were performed by illuminating the cells at 340 and 380 nm. Emitted light was filtered through a 510 nm filter and detected by a camera. The cells were superfused with the solutions of interest and data was recorded using MetaFlour software.

### Statistical analyses

The number of independent experiments are indicated in the legends to Figures; values shown are the mean±SEM, with significance (p<0.05) determined by repeated measures or one-way ANOVA, followed by Dunnett or Tukey-Kramer post t- tests, as appropriate. P values are for the comparisons indicated.

## SUPPLEMENTARY MATERIALS FIGURES


