# Supplementary material for: RPGR protein complex regulates proteasome activity and mediates store-operated calcium entry
Source: Oncotarget. 2018 May 1;9(33):23183–97. doi: 10.18632/oncotarget.25259 (PMC5955404; doi:10.18632/oncotarget.25259)
Supplement: Supplementary file 1 [file oncotarget-09-23183-s001.pdf]

RPGR protein complex regulates proteasome activity and mediates store-operated calcium entry

SUPPLEMENTARY MATERIALS

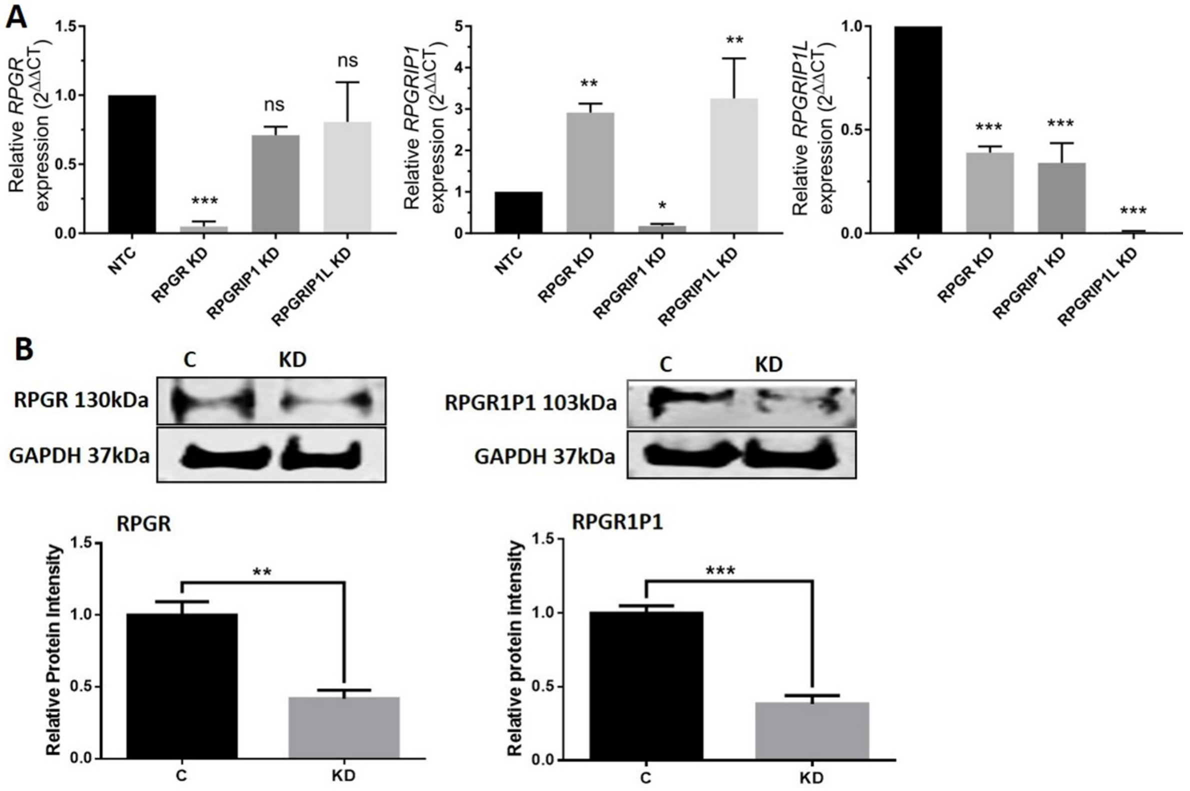

Supplementary Figure 1: (A) mRNA expression levels of *RPGR, RPGRIP1* and *RPGRIP1L* in scrambled control (C), *RPGR, RPGRIP1*, or *RPGRIP1L* knockdown (KD) in hTERT-RPE1 cells as determined by qPCR analysis. mRNA level of *RPGR, RPGRIP1* or *RPGRIP1L* was significantly down regulated in the KD cells when compared the control cells. It is interesting that knockdown of *RPGRIP1* caused changes in *RPGR* and *RPGRIP1L* expression, and knockdown of *RPGRIP1L* also caused changes in *RPGR* and *RPGRIP1* expression. However knockdown of RPGR did not cause any change in either *RPGRIP1* or *RPGRIP1L* expression. The data was normalized to the *GAPDH* gene. **(B)** Western blot showing the level of RPGR or RPGRIP1 was significantly decreased in RPGR or RPGRIP1KD cells, respectively. 57.47% reduction of RPGR protein in *RPGR* knockdown cells and 62.01% reduction of RPGRIP1 protein in *RPGRIP1* knockdown cells when compared the control cells. Both mRNA and protein levels in control and RPGR or RPGRIP1 knockdown cells were detected at 48h after transfection, it is possible RPGR and RPGRIP1 proteins have quite long half-life and led to about 42.53% of RPGR protein and 37.99% protein remained, though quite lower levels of RPGR and RPGRIP1 mRNA were detected. The band intensities of RPGR, RPGRIP1 and GAPDH were quantified, for comparison, the intensity of RPGR or RPGRIP1 was normalized to the intensity of GAPDH, the ratio of RPGR to GAPDH or RPGRIP1 to GAPDH in scrambled control (C) was regarded as 1.0. Data were from three independent experiments. $^{*}p<0.05$; $^{**}p<0.01$; $^{***}p<0.001$.

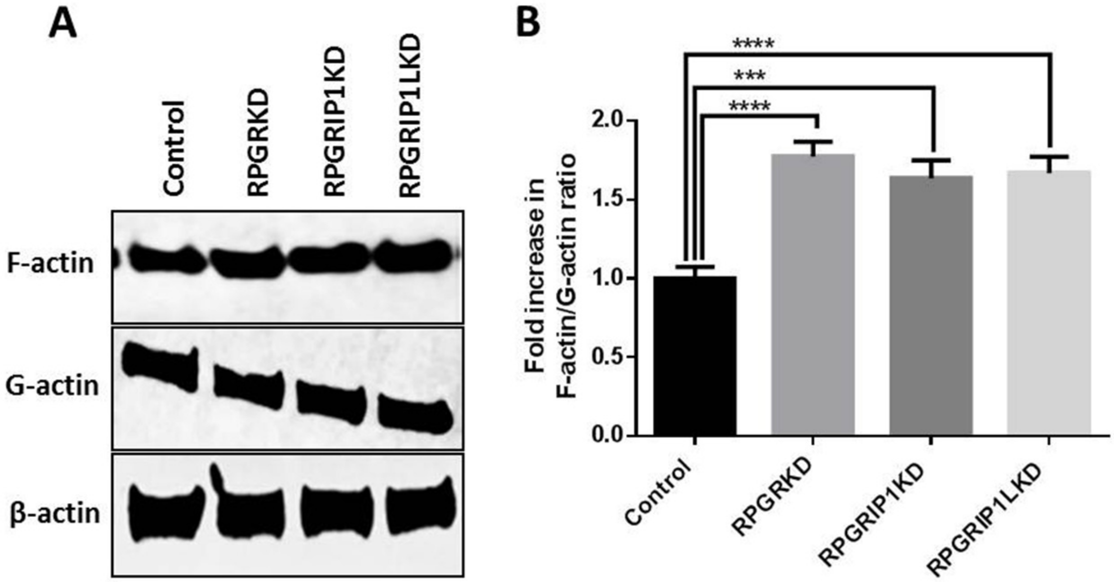

Supplementary Figure 2: Actin cytoskeletal defects in *RPGR, RPGRIP1* and *RPGRIP1L* knockdown (KD) hTERT-RPE1 cells. (A) F-actin and G-actin were fractionated according to the description in Materials and Methods. The fractionated F-actin and G-actin of the control and KD cell lysates were analyzed by immunoblotting using anti-β actin antibody. Equal amount of total protein was subject to immunoblotting as control for equal actin in control and KD cell lystaes. **(B)** The ratio of F-actin to G-actin was analyzed by the band intensity in the F-actin and G-actin fractions of control and *RPGR, RPGRIP1* or *RPGRIP1L* KD cells. The ratio of F-actin to G-protein in control cells was regarded as 1.0. Data were from three independent experiments. $^{***}p<0.001$; $^{****}p<0.0001$.

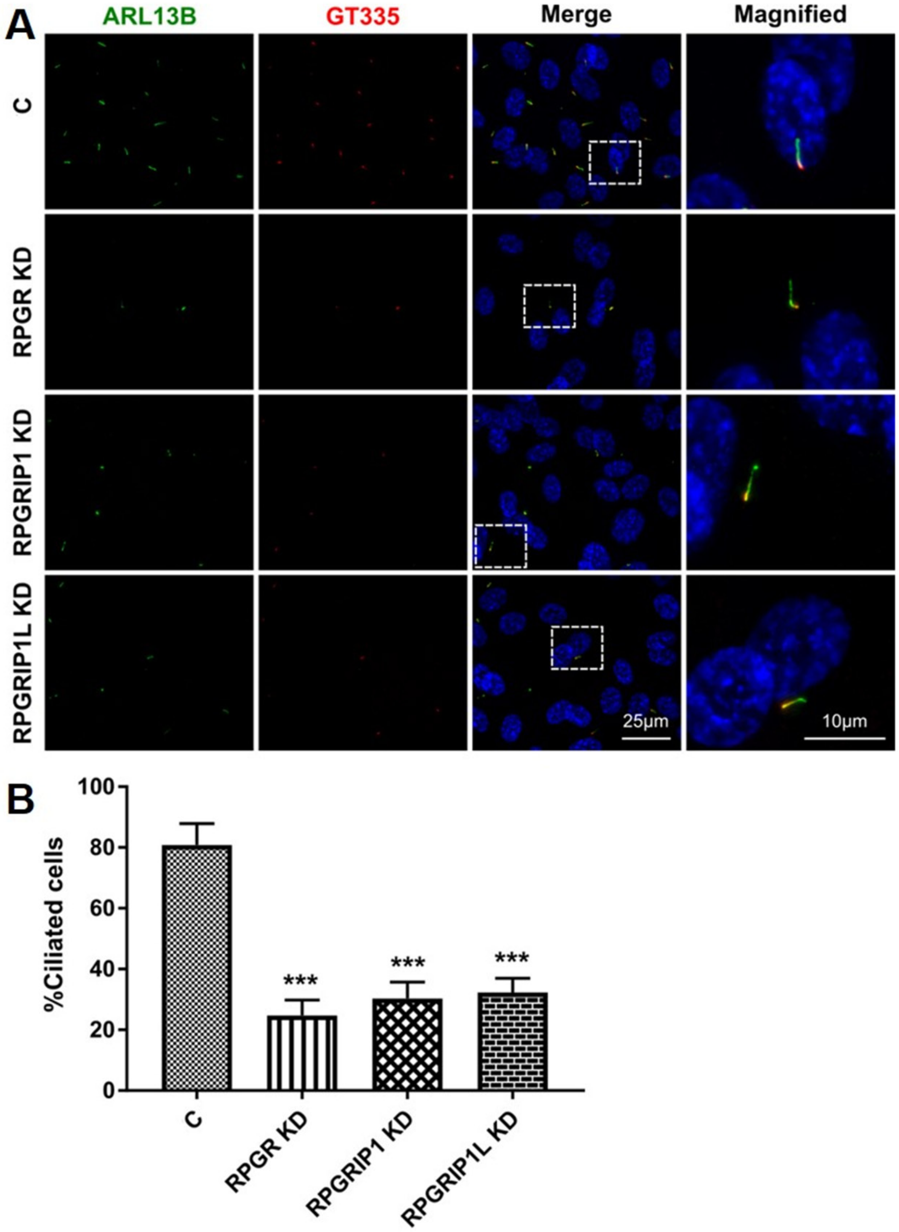

Supplementary Figure 3: Knockdown (KD) of *RPGR, RPGRIP1*, or *RPGRIP1L* impaired ciliogenesis. (A) hTERT-RPE1 cells were transfected with control (C), *RPGR, RPGRIP1*, or *RPGRIP1L* specific siRNAs. Forty eight hours after transfection cells were serum-starved for 48h and then cilia formation was analyzed using anti-ARL13B (axoneme marker, green) and GT335 (basal body/ transition zone marker, red). DAPI was used to visualize nuclei. Cells with green and red signals were ciliated. Magnified images show a 4× magnification of the selected regions. **(B)** Graph showing decreased cilia number in *RPGR, RPGRIP1*, or *RPGRIP1L* KD cells when compared to the control (C). Data were presented as means±SEM, statistical significance was analyzed using one-way ANOVA followed by the Dunnett's multiple comparison tests. Data were from two independent experiments. In each experiment, number of ciliated cells in total 150 cells (control and specific siRNA KD) was counted. ***p<0.001.

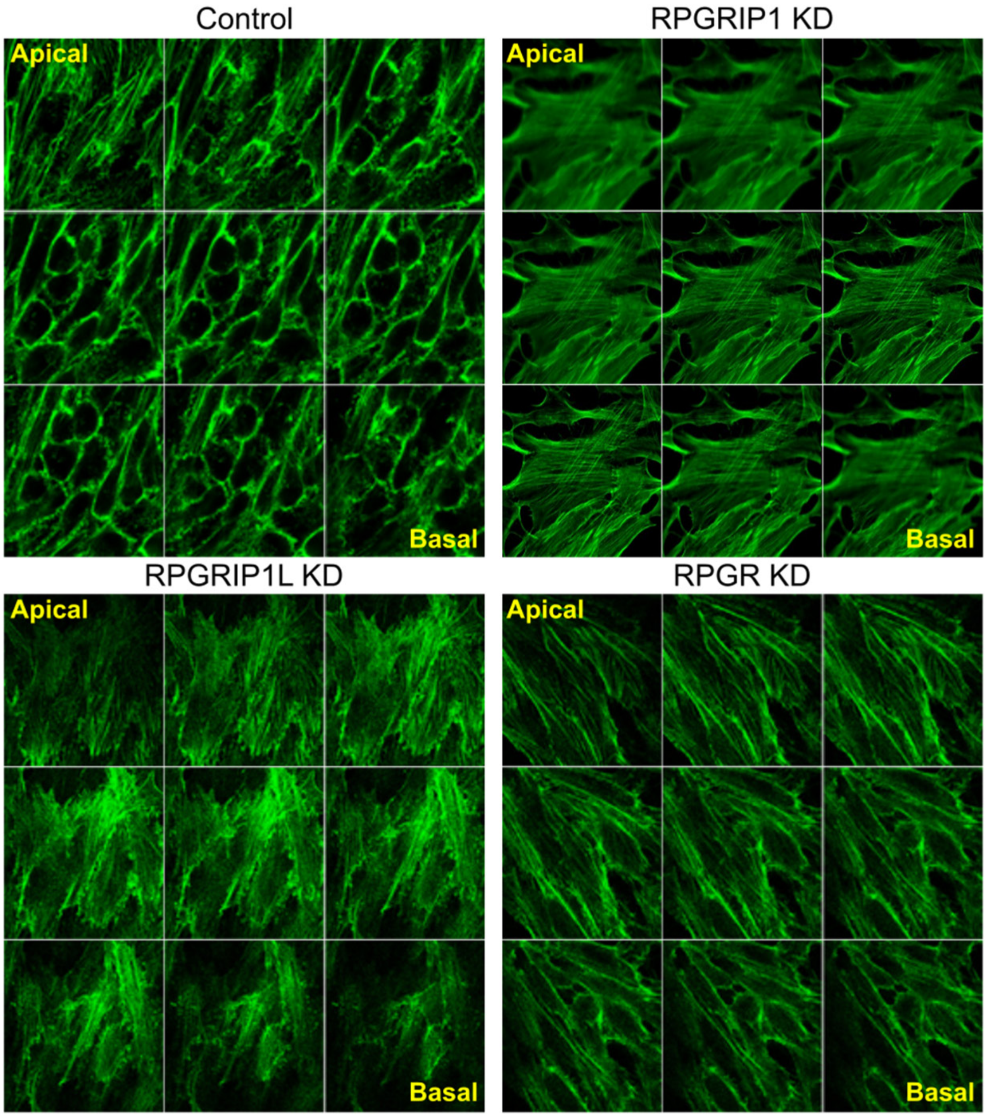

Supplementary Figure 4: Z-stacks images showing stronger actin filaments in RPGR, RPGRIP1 or RPGRIP1L silenced hTERT-RPE1 cells. hTERT-RPE1 cells were transfected with scrambled control or RPGR, RPGRIP1 or RPGRIP1 siRNA for 48h and then serum starved for 48h, followed by FITC-conjugated phalloidin staining.

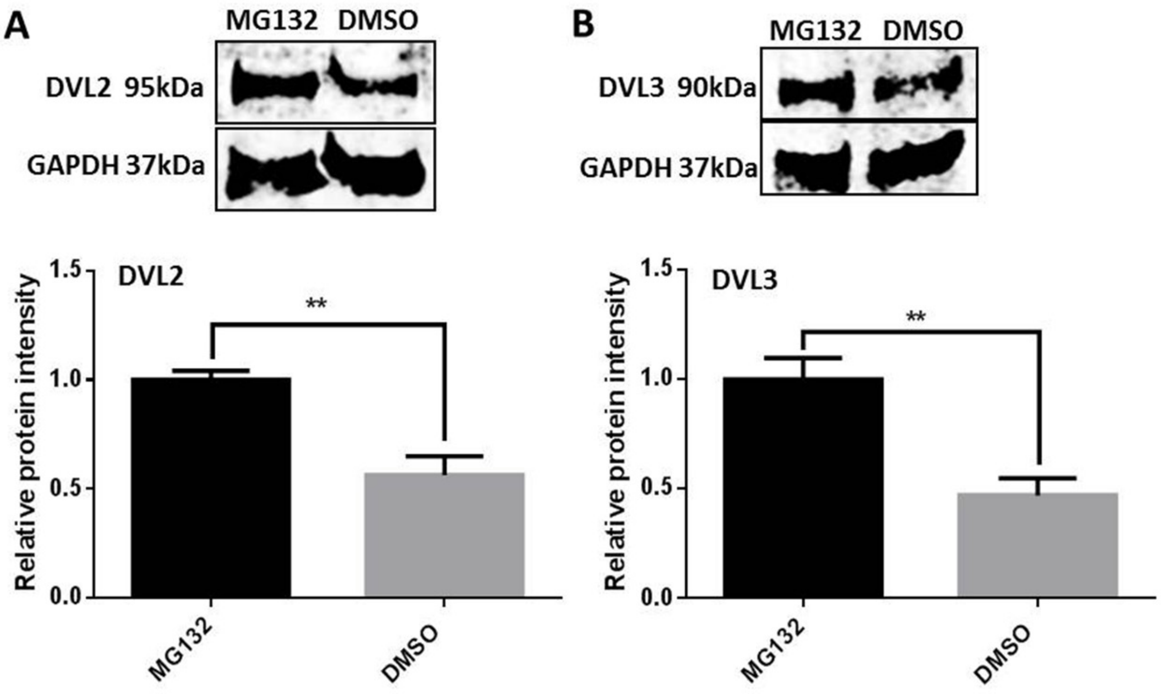

Supplementary Figure 5: Significantly increased levels of DVL2 and DVL3 in hTERT-RPE1 cells treated with MG132. **(A)** hTERT-RPE1 cells were treated with MG132 (40µM) for 24 hours, control cells were teated with DMSO. DVL2 and DVL3 in cell lysates were detected by Western blotting using anti-DVL2 and anti-DVL3 antibodies. The bolts were reprobed with GAPDH as loading controls. **(B)** Graph represents the relative band intensity of DVL2 and DVL3 in control and MG132-treated cells. The band intensities of DVL2 and DVL3 were measured and normalized with the band intensity of GAPDH. The ratio of DVL2 or DVL3 to GAPDH in scrambled control cells was regarded as 1.0. The data were represented as means±SEM. The experiment was repeated three times. $^{**}p<0.01$.

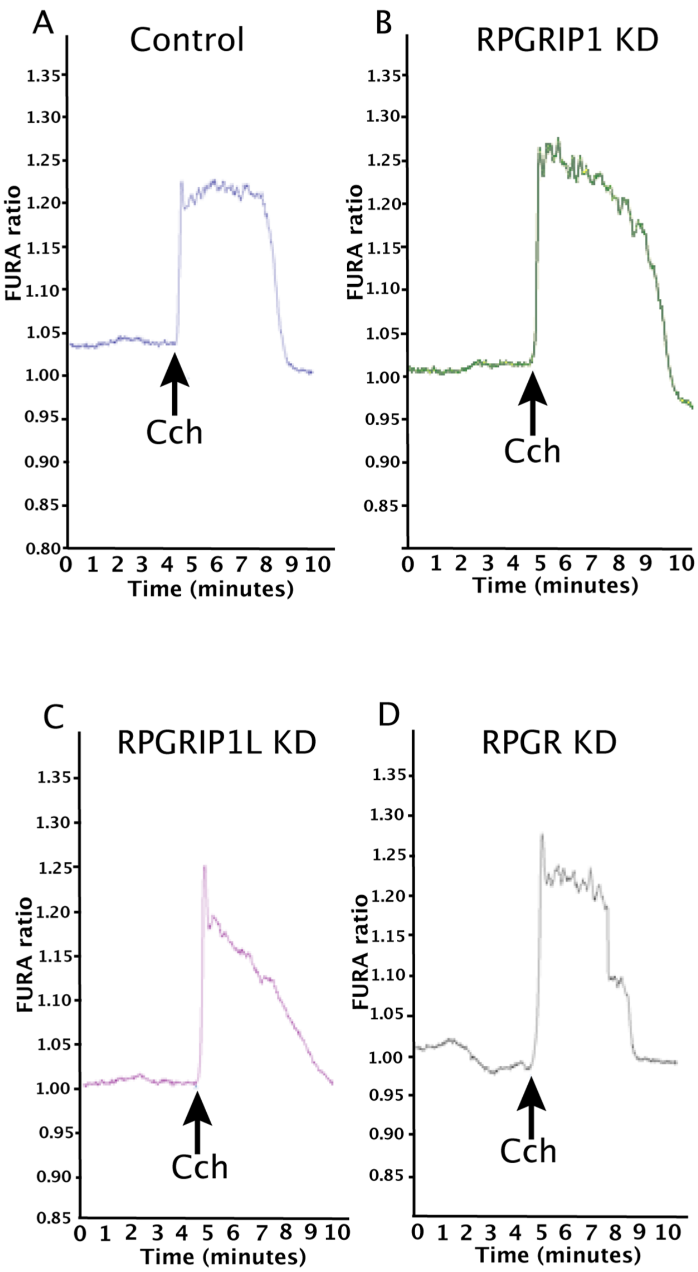

Supplementary Figure 6: Effect of Carbachol (CCh) on store calcium release: Control **(A)** and knockdown (KD) hTERT-RPE1 cells **(B, C** and **D)** in Ringer solution with Ca^{2+} were exposed to 10μM CCh in Ca^{2+} free solution followed by a Ca^{2+} free wash. The release of Ca^{2+} from the store in response to CCh – ie the peak Fura-2 ratio - was similar in control and KD hTERT-RPE1 cells.
